# Democratic Policing and Officer Well-Being

**DOI:** 10.3389/fpsyg.2020.00874

**Published:** 2020-05-26

**Authors:** Kimberly C. Burke

**Affiliations:** Department of Sociology, Center for Policing Equity, University of California, Berkeley, Berkeley, CA, United States

**Keywords:** police, officer well-being, democratic policing, procedural justice, community-oriented policing, us versus them, occupational stress

## Abstract

Efforts to improve police–community relationships have increased initiatives that aim to build trust and mutual respect between officers and the communities they serve. Existing literature examines the impact of internal departmental dynamics and individual-level characteristics on officers’ endorsement of community-oriented policing strategies. Research has indicated that when officers feel fairly treated within their agencies and when they are less psychologically and emotionally distressed, they report stronger support for policing tactics that increase fairness in police processes and decision making. This mixed-method study is the first to examine the reciprocal relationship by asking: How do procedurally just and community-oriented policing strategies impact officer well-being and occupational stress? Sworn officers in a medium-sized California department completed a survey assessing their views on their agency, various police tactics, the communities they serve, and their physical and mental health. Results showed that officers’ increased support for community-oriented and procedurally just police strategies are significantly associated with decreased job stress, depression, anxiety, and negative affect, controlling for race, gender, perceived job dangerousness, cynicism, and how many years they had served as a police officer. In-depth interviews with officers in the department revealed three explanatory mechanisms for these statistical relationships. First, the tenets of procedural justice provided officers with tactics that reduce the threat and stress of intergroup interactions. Second, community-oriented policing activities increased opportunities for officers to have positive interactions with the communities they work in, mitigating the distrust, cynicism, and detachment fostered by enforcement activities. Last, procedural justice and community-oriented police strategies empowered officers to counter negative stereotypes about police and reaffirm their self-image. Taken together, these survey and interview findings highlight the mutuality of democratic policing and officer wellness.

## Introduction

There exist countless efforts to build trust and mutual respect between officers and the communities they serve. Many of these efforts are grounded in a philosophy of democratic policing that shifts away from a narrow focus on enforcement and deterrence toward policing efforts that build public trust. Democratic policing styles focus on establishing legitimacy through procedural justice, which is rooted in fair and respectful treatment, as well as community-oriented policing programs that are community focused and participatory (Trinkner et al., 2016). While institutional aims to build trust and mutual respect between officers and the communities they serve are ostensibly admirable, many fall short in part because they are built absent the input of the very workers charged with implementing and enforcing those mandates—patrol officers. Evidence suggests that patrol officers (those who work in uniform and/or in the field) experience routine occupational discontent emergent from failed or poorly drafted attempts to improve police–community relationships (Violanti et al., 2006, 2007; Ma et al., 2015). A lack of trust and cooperation can engender more stressful interactions between police and communities of color. For example, interview data collected from almost 8,000 officers in over 50 departments across the United States, revealed that 87 percent of officers in large departments report more tense interactions between police and Black civilians as a result of high-profile violent incidents involving Black victims and police (Pew Research Center, 2017). These findings reflect the “us versus them” dynamics that permeate police culture. “Us versus them” describes police officers’ feelings of isolation from the general public resulting from negative portrayals of police in the media, high-profile incidents of police misconduct, and negative police–civilian interactions that foster a divide between police and the communities they serve (Brough et al., 2016; Boivin et al., 2018). “Us versus them” attitudes in policing negatively impact public safety by reducing communities’ willingness to cooperate with police by reporting crimes. Communities of color are less likely to cooperate with police than White communities due to mistrust stemming from experiences of racial profiling, police use of excessive force, and disparities in the enforcement and consequences of drug laws (Tyler, 2005).

In addition to harming public safety, “us versus them” dynamics present a source of chronic occupational stress for officers. A growing body of literature examines how occupational stress shapes officer well-being and their capacity to police safely. It has been shown that officers experiencing chronic stress demonstrate slower reaction times and weakened verbal memory, which directly impacts their ability to perform their duties effectively (Papazoglou and Andersen, 2014). Research has also indicated that when officers are less psychologically and emotionally distressed, they report a stronger endorsement of democratic forms of policing (Tyler and Fagan, 2008; Trinkner et al., 2016). Another study found that surveyed officers who are concerned with appearing racist when interacting with community members tend to both report diminished confidence in their legitimate authority and endorse coercive policing tactics (Trinkner et al., 2019). When officers are less stressed, they engage in more democratic policing that has been linked to increased public safety. In contrast, less emotionally and psychologically healthy officers experience adverse effects on their work performance that necessarily harms the communities they police (Andersen and Papazoglou, 2015).

**FIGURE 1:**
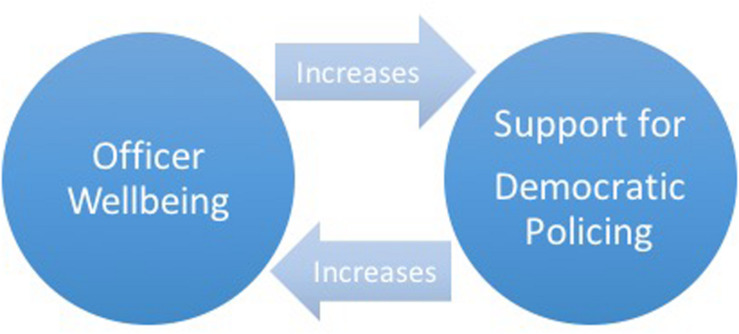
The mutuality of officer well-being and democratic policing.

Existing research examines why emotionally healthy officers endorse and engage in positive policing practices and suggests that healthy officers only remain healthy given opportunities to police democratically. Together, these findings make explicit the inextricable relationship of officer and community wellness. I designed this mixed-methods study to address the mutuality of officer and community wellness (Figure 1). This study examines how positive policing practices may reciprocally impact officer well-being and shape their experiences of “us versus them” dynamics. I ask: What is the relationship between officer well-being, democratic policing, and “us versus them” dynamics? This study provides patrol officers’ accounts of democratic policing efforts and their experiences working in civilian communities with tense police–community relationships. Drawing on officers’ self-reported descriptions of their support for democratic policing, I explore the implications of democratic policing for officers’ navigation of “us versus them” dynamics and the relationship to officer well-being.

## Materials and Methods

### Sample and Recruitment Strategy

This cross-sectional mixed methods study uses survey and interview data from a convenience sample of sworn officers from the patrol division of the California Police Department (CPD), a midsized agency that employs over 500 sworn officers and less than 200 civilian personnel to police a population of around 300,000. The agency is composed of around 60 percent White officers and 40 percent officers of color while the population of the city was approximately 20 percent White and 80 percent people of color with Latinx comprising over 20 percent of the population.

### Survey Data and Methods

Survey data were collected by the Center for Policing Equity (CPE) where I am a research affiliate. Survey participants were recruited in 2018 through announcements made during CPD’s roll calls, the supervisor-led briefings that occur before each group of officers deploys for their morning, afternoon, and night shifts. At CPD, all officers deployed from headquarters as opposed to distinct and separate precincts. Surveys were administered through an online survey platform, and officers were permitted to take the surveys on duty and instructed to skip any questions or sections they so choose. There were 86 total respondents yielding a response rate of 17 percent. Of the 86 respondents who completed the survey, only 67 provided complete data. Cases of missing data were fairly evenly spread across variables. Of the 67 respondents with complete data, 91 percent were men (*n* = 61), 9 percent were women (*n* = 6) and a little over half were White, 51 percent (*n* = 40). These sample characteristics are similar to the demographic makeup of CPD. On average, officers reported 9.35 years of experience as a police officer (SD = 6.78).

All items used 7-point unipolar response options and were coded so that higher scores indicated a greater amount of what was being measured. Scales were constructed by averaging the items and were generated in Stata using the “alpha” command which reports Cronbach’s alpha measure of the reliability of each scale (Croasmun and Ostrom, 2011). The accepted standard for alphas across social sciences is 0.70, which all of the scales used in this study exceed. Reliability coefficients and descriptive statistics for all measures are shown in Table 1.

**TABLE 1:** Descriptive statistics.

	*M*	*SD*	*Min*	*Max*	*A*
Democratic Policing	6.09	0.94	3.22	7	0.90
**Officer Well-being**					
Job stress	3.70	1.16	1	6.5	0.83
DASS	1.40	0.44	1	3	0.94
Negative affect	2.17	0.83	1	5	0.87
**Controls**					
Cynicism	3.41	1.21	1	5.75	0.77
Job dangerousness	6.22	2.34	1	7	
**Demographics**					
Years of experience	9.35	6.78	1	23	
Race	White: *n* = 40, 51%		Non-White: *n* = 27, 49%		
Gender	Men: *n* = 61, 91%		Women: *n* = 6, 9%		

**N* = 67.*

I operationalized officer well-being using three continuous outcome variables: occupational stress; depression, anxiety, and stress (DASS); and negative affect.

The first indicator of officer well-being is a four-item occupational stress scale (Lambert et al., 2007) that asks officers how they feel when they are at work generally, as opposed to how they felt at a specific moment in their life course (e.g., “How often do you feel tense or uptight at work”) and captures responses on a 7-point Likert scale from 1 = “Never” to 7 = “Always.”

The second indicator of officer well-being is a 21-item measure (Lovibond and Lovibond, 1995) that asks participants to focus on how often they experienced symptoms of depression, anxiety, and stress (DASS) within the past 6 months (e.g., “I couldn’t seem to experience any positive feeling at all”) with responses on a 7-point Likert scale from 1 = “Never” to 7 = “Always.”

The third indicator of officer well-being is a 14-item measure adapted from the positive and negative affect scale (Watson et al., 1988) and reverse coded to provide a measure of negative affect. This scale consists of a number of words and phrases that describe different feelings and emotions (like nervous, angry, and alert) and asks participant to indicate to what extent they have felt this way in general, as opposed to how they felt at a specific moment in their life course. Responses are captured on a 7-point Likert scale from 1 = “Very slightly or not at all” to 7 = “Extremely.”

Officers’ endorsement of democratic policing is a nine-item scale that measured officers’ support for a community-oriented model of policing (e.g., “Community-oriented policing keeps the community safe”) and support for process-based policing grounded in the tenets of procedural justice (e.g., “How important is to allow community members to voice their opinions when you interact with them?”) (Trinkner et al., 2016). Previous research measuring support for democratic policing has distinguished between the endorsement of community-oriented policing and endorsement of procedurally just policing. In this sample, those constructs are strongly correlated, so I collapsed them into a single scale that has a reliability coefficient of 0.90.

I controlled for the gender and race of the officer and how many years they had served as a police officer. Officers reported their job experience by indicating how many years they had served as a police officer. I accounted for three additional variables that could have potentially influenced the outcomes of officer well-being. Cynicism compounds experiences of stress, necessarily impacting officer well-being. Officers with more experience on the job and/or greater experiences of dangerous and traumatic incidents may also be more likely to have negative mental health outcomes. To control for these effects and to help isolate the association of support for democratic policing from the risks inherent in the policing profession, I accounted for perceived job dangerousness and cynicism. Officers were asked “How dangerous is your job?” with responses captured on a 7-point Likert scale from 1 = “Not at all” to 7 = “Extremely.” Cynicism is four-item measure that assesses officers’ negative and apathetic views about the communities they police (e.g., “Community residents are willing to help the police identify criminals”) with responses captured on a 7-point Likert scale from 1 = “Strongly disagree” to 7 = “Strongly agree.”

### Interview Data and Methods

I collected interview data from a convenience sample of patrol officers and sergeants from the patrol division of a CPD. The interview sample was composed of 15 respondents, ranging from 1 to 22 years on the force, with demographics reflective of the department: 10 White, 2 Black, 1 Latinx, 2 Asian, 13 men, and 2 women. I completed interview recruitment in the fall of 2018. Participants were recruited via email announcements sent to their departmental emails from leadership. I also spent 3 days recruiting during CPD’s roll calls. During roll calls, I asked officers interested in conducting hour-long one-on-one interviews about de-escalation tactics to provide their names and phone numbers on sign-up sheets. I scheduled and conducted all the interviews. Eight of the interviews were conducted in a hotel meeting area near the police headquarters, four were conducted in private conference rooms at the police department, and three were conducted over the phone. Officers were permitted by leadership to complete interviews while on duty. Interviews ranged from 1 to 2 h and were digitally recorded, transcribed verbatim, and imported into the Dedoose online software for coding. Interviews were coded iteratively by myself and a research assistant, following the mandates of abductive analysis (Timmermans and Tavory, 2012). We created our initial codes based on the interview protocol (Appendix A) and policing literature and then with the themes that emerged in the data.

I conducted this study after more than 4 years of having worked with police departments across the nation in my capacity as a project director at CPE. CPE addresses issues of racial bias in policing using a contemporary understanding of bias in which stereotypes can influence perceptions and behaviors outside of an individual’s conscious awareness (Burke, 2020). CPE’s research counters the popular “bad apples” framing in police reform efforts that “suggests the racist attitudes of a handful of officers cause racially biased policing” and instead “foregrounds the understanding that racially biased policing outcomes, like racial disparities in use of force, can occur in the absence of explicit or conscious racial animus” (Burke, 2020: 37). I designed this study with that framework in mind and constructed interview questions that would determine if different officer characteristics mapped onto different understandings of democratic policing strategies.

I organized interview respondents into Warrior/Guardian typologies based on prevailing scholarship that suggests that officers with Warrior mindsets have a different orientation to police tactics than their more egalitarian Guardian colleagues (Paoline and Gau, 2018; Carlson, 2019; McLean et al., 2019). Officers classified as Warriors focused on fighting crime and upholding the law and viewed policing as the thin blue line between good and evil and social order and chaos as the most important aspects of policing. Further, officers in the Warrior category considered police work exciting and adrenaline inducing. The attitudes of officers categorized as Warriors are exemplified in the following excerpts:

“[Being a police officer means] being able to go out and help people and catch the bad guys and hopefully keep guns and drugs off the street…” (Officer 1, White man, Warrior).

“I’m kind of like an adrenaline junkie and I want to be the person out there chasing these guys with guns” (Officer 11, White man, Warrior).

The Warrior cop is juxtaposed with the Guardian cop, defined by a commitment to building trusting relationships with community members and protecting those members. Guardian officers emphasized service and helping members of the community more than fighting crime. The mindsets of officers in the Guardian category are highlighted in the following excerpts:

“[the most important part of police work is] … helping people who need help…” (Officer 8, White man, Guardian).

“… being able to help the people who can’t help themselves. Children, women, elderly. I mean, men too… And sometimes you’re just a helping hand to get them out of whatever situation they’re in. I try to make sure I do that every day” (Officer 6, Asian man, Guardian).

I classified a total of seven interview respondents as Warrior cops with the following demographic breakdown: six White, one Black, six men, and one woman. I classified a total of eight interview respondents as Guardian cops with the following demographic breakdown: four White, one black, one Latinx, two Asian, seven men, and one woman. Full demographic breakdowns are provided in Table 2.

**TABLE 2:** Warrior/Guardian typology.

	Warrior	Guardian
Race	White: *n* = 7	White: *n* = 1, Asian: *n* = 2, Black: *n* = 2, Latinx: *n* = 2
Gender	Men: *n* = 6, women: *n* = 1	Men: *n* = 7, women: *n* = 2
	*n* = 7	*n* = 8
		

## Findings

### Survey Data

I analyzed all survey data using multivariate regression models with a one-tailed test of significance. The beta coefficients from the models examining officers’ support for democratic policing and occupational stress are reported in Tables 3–5 where the first models (M1) are the bivariate relationship between support for democratic policing and the outcome variables (occupational stress, DASS, and negative affect); the second models (M2) control for officer race, gender, and years of experience as a police officer; the third models (M3) control for the demographics listed in M2 and perceived job dangerousness; and the fourth models (M4) add in the control measure of cynicism to M3. Across all models, the data suggest that endorsement for democratic policing is associated with a statistically significant decrease in occupational stress, DASS, and negative affect.

**TABLE 3:** Support for democratic policing and occupational stress.

	M1: Occupational Stress	M2: Demographics	M3: Job dangerousness	M4: Cynicism
Support for democratic policing	−0.370**	−0.357**	−0.296*	−0.229*
	(0.147)	(0.148)	(0.138)	(0.141)
*R*-squared	0.089	0.197	0.331	0.364

*N* = 67. Standard errors in parentheses. *p < 0.05, **p < 0.01, ***p < 0.001.

**TABLE 4:** Support for democratic policing and DASS.

	M1: DASS	M2: Demographics	M3: Job Dangerousness	M4: Cynicism
Support for democratic policing	−0.206***	−0.219***	−0.202***	−0.199***
	(0.052)	(0.054)	(0.052)	(0.055)
*R*-squared	0.089	0.197	0.331	0.364

*N* = 67. Standard errors in parentheses. *p < 0.05, **p < 0.01, ***p < 0.001.

**TABLE 5:** Support for democratic policing and negative affect.

	M1: Negative affect	M2: Demographics	M3: Job Dangerousness	M4: Cynicism
Support for democratic policing	−0.419***	−0.433***	−0.410***	−0.397***
	(0.097)	(0.101)	(0.100)	(0.105)
*R*-squared	0.223	0.272	0.312	0.314

*N* = 67. Standard errors in parentheses. *p < 0.05, **p < 0.01, ***p < 0.001.

### Interview Data

Survey data support the idea that endorsement of democratic policing and officer well-being are positively associated. However, these survey results taken alone do not explain how or why these forms of democratic policing relate to officers’ mental health. I identified three key themes in the interview data that help explain the association between endorsement for democratic policing strategies and reduced stress and negative affect. Each of these themes foregrounds the salience of “us versus them” as a source of stress in officers’ daily work and emerged in the context of officers discussing tense or stressful interactions with community members, specifically in how they navigate and/or overcome negative stereotypes. First, officers described relying on strategies that are rooted in the tenets of procedural justice in order to navigate and reduce the stress of intergroup interactions. Second, they described community-oriented policing activities, defined as non-enforcement-related activities, as providing them with opportunities to have positive interactions within the communities they serve. Last, there was a connection between their deployment of procedural justice and their engagement in community-oriented policing and a reaffirmation of a positive self-image. Importantly, I identified these themes across the Warrior and Guardian typologies and found that officers who demonstrated a Warrior mindset were equally likely as those with Guardian mindsets to describe service and non-enforcement community interactions as central to their job. This finding indicates the importance of department-level standards around democratic policing strategies, perhaps even above and beyond individual-level characteristics.

Social psychological research indicates that individuals often experience intergroup interactions as threatening and stressful. This is particularly true for officers due to their concerns about negative assessments by community members (Pew Research Center, 2017; Trinkner et al., 2019). The first theme I identified is that officers in this study sample reported their ability to reduce tension in police–civilian interactions by using procedurally just policing strategies. For example, Officer six exemplified neutrality and respect when describing his interactions with people who have been constructed as criminals:

“…just because they’re in a gang or they have a criminal record, they’re still people… We deal with the one percent, and that’s the one percent. It’s the ones that are always committing violence, they’re always committing crimes, or the gang bangers, the drug users, the drug dealers. We’re always dealing with them, but in the end, they can be a suspect 1 day and a victim the next day. We can’t just treat them like a suspect all the time. We have to treat them like people regardless of what they do… You show people respect, you get the respect” (Officer six: male, White, 3.5 years of service, Warrior mindset).

Similarly, Officer five, who grew up in the neighborhood he now polices, cited his use of transparency to facilitate positive interactions:

“By going the extra mile to explain to them, you know, this is why this happened… This is the reason why I’m giving you this ticket, or this is the reason why we had to pull our guns out. Whatever the situation is, I try to make sure that I allow myself to explain that. And I think once they see that, they understand like, oh yeah, he’s a cop, but he actually cares. He’s willing to go that extra mile to explain to me what’s going on. He’s treating me as a human being basically… You treat people right, nine times out of ten they’ll treat you right back” (Officer three: male, Black, 5 years of service, Guardian mindset).

Other officers discussed the value of listening and creating space for people to express their experiences and frustrations. By treating people with neutrality and respect, centering their shared humanity, giving them voice, and being transparent in their decisions, these officers reported having more positive, less tense interactions. In this way, their use of procedural justice mitigated “us versus them” stressors.

The second theme highlights how community-oriented policing activities increased opportunities for officers to have positive interactions with the communities they serve. Community-oriented policing is a phrase that covers a diversity of practices. For this department, community-oriented policing was consistently described as the non-enforcement interactions that officers have with community members. Police are asked to deal with people on their worst days. As alluded to by Officer 6 in the above quote, police mostly interact with “the one percent,” a small percentage of the population that is in crisis, as either perpetrators or as victims of crime. Research shows that when these interactions form the entirety of police activities, officers can come to view that small percentage as representing all of humanity, which fosters a negative worldview that makes them more likely to have hostile interactions with civilians (Regoli et al., 1990). Officers in this study described community-oriented policing in ways that suggest its potential for reducing negative affect fostered by enforcement-only interactions. For example, Officer 9 described community-oriented policing as giving him an opportunity to have positive and meaningful engagements with members of the community:

“I actually talk to them and get more into their background and understanding what was going on … You have the time to have one-on-one interactions with people again and you start getting past the biases that you develop over time…” (Officer nine: male, White, 22 years of service, Guardian mindset).

Importantly, these engagements were viewed as an integral part of police work, rather than an isolated element assigned only to a specific community-focused unit, as exemplified by Officer one:

“Without community engagement and talking with people and trying to lighten their day up, there’s no police force really, because if every single citizen out there was against us, then how are we ever gonna be able to do the job that we signed up to do, and I tell people there’s just no way we could, we couldn’t do it” (Officer one: male, White, 1 year of service, Warrior mindset).

These data suggest that community-oriented policing that facilitates non-enforcement interactions may counteract, or balance, the negatively biased outlook engendered by enforcement-related police work.

The third and final link between officer wellness and democratic policing relates to their self-image. Studies have shown that self-image is strongly related to depressive symptoms (Erkolahti et al., 2003). Overwhelmingly, officers in this study described themselves as “good people” and as “protectors.” These descriptions were used across the Warrior/Guardian typologies as illustrated by Officer 4:

“We have to take care of everyone. We’re warriors of our own class. That may sound silly, but it’s true. Because no matter what, we have to protect the women, the children, we even have to protect the bad guys, believe it or not” (Officer four: female, White, 27 years of service, Warrior mindset).

When asked about stereotypes of police, all 15 interview respondents described having to contend with negative stereotypes in police–civilian interactions. Research shows that when officers are concerned with appearing racist, they report diminished confidence in their self-legitimacy and are more likely to rely on coercive tactics (Trinkner et al., 2019). However, Officer four described using the tenets of procedural justice to navigate accusations of racism that reaffirm her self-legitimacy and self-image as a protector. She recounted an incident involving a vehicle stop where the driver accused her of racial profiling. Officer four described first listening to the driver’s complaints and then explaining her decision making by clarifying the violation that prompted the stop and sharing with the driver that she had not been able to see him when she made the decision to pull him over. By taking the time to listen to the driver, validate his concerns, and share her perspective, Officer four was able to reinforce her positive self-image in the face of negative stereotyping.

Similarly, Officer five described relying on the procedural justice principles of giving voice and practicing neutrality in his dialogue with community members to counter their negative stereotypes of police, providing him the opportunity to affirm a positive self-image:

“Whatever it is that’s causing them to be frustrated, upset, non-compliant, because they’re frustrated at the world, or police… There’s a lot of things you can solve just by getting people to talk. And give them some sort of thing to relate to such as race … place of birth, that I grew up here, I’m one of you guys. I tell them I’m just a normal person. You know? A thing people say a lot is oh, you’re a cop. You know, take that badge off and you’re nothing. You’re right, I’m not. I’m just a dude like you are. I am just a guy… I’m just doing a job you know? …it’s just a whole different level of communication when they feel like you can relate to them versus when you’re just the cop here to do, you know, knock people’s skulls together and take people to jail” (Officer five: male, Asian, 3 years of service, Guardian mindset).

## Discussion

Police work is characterized by dangerous and distressing interactions that can create maladaptive coping behaviors that contribute to experiences of stress (Russo et al., 2020). Routine occupational stress exposure presents a significant risk factor for psychological distress among police officers (Liberman et al., 2002). The current study identifies the ways in which support for and engagement in democratic policing can positively impact officer well-being by reducing the stress of “us versus them dynamics,” mitigating negative affect through positive engagements with communities, and providing officers opportunities and tools to reaffirm a positive self-image. These findings are consistent with evidence from an experimental study which found that officers were less likely to resolve incidents with an arrest or a use of force following “a procedural justice training program designed to “slow down” police officers’ thought processes during citizen encounters” (Owens et al., 2018).

As I suggested in my findings, different types of officers in these data coalesced in their understandings of procedural justice and community-oriented policing which indicates a department-level standard around these strategies. Many respondents explicitly described democratic policing as a departmental value. For example, one officer described non-enforcement community engagement as a leadership priority:

“I think from the chief down and what he’s trying to implement, I think he’s trying to implement that we’re out here to actually help people, not just be that hammer of the law.”

Another officer described fair treatment as part of a departmental culture that values neutrality in decision making, even when someone has committed a crime:

“… we try to treat [people] fairly… it’s pretty much how the police chief has pretty much formed this department …to treat people fairly and not judge them for their actions. ‘Cause everybody makes mistakes from time to time, no matter who you are, rich, poor, right? You can be the dumbest person and the smartest person but still make the same mistake…”

During data collection for this study, this department was nearing the end of their participation in a 4-year pilot program aimed at increasing community trust. As part of this initiative, the department conducted trainings on procedural justice and bias reduction and held a series of “listening sessions” aimed at racial reconciliation, the results of which informed policy changes including updated general orders and officer evaluations with measures of procedural justice. Community surveys were conducted at the start (Wave 1) and end (Wave 2) of the pilot program by an outside research partner and were completed by almost 200 residents in the jurisdiction’s neighborhoods with the highest indices of concentrated crime and poverty (the pilot study and research center names have been removed to protect confidentiality and adhere to institutional review board standards). These surveys revealed statistically significant increases from Wave 1 and Wave 2 in community respondents’ perceptions of police use of procedural justice, community-focused policing, relatability to police, and willingness to partner with police. This department demonstrated its commitment to procedural justice and community-oriented policing and valued it enough to incorporate it into officer evaluations. In doing so, patrol officers across a range of Guardian/Warrior mindsets exhibit similar valuations of democratic policing styles. Summarily, this study suggests department-specific findings and maps onto a conversation about what police departments can and should do to build an infrastructure that more effectively supports officer health and community safety.

I acknowledge that relying on a convenience sampling method does not yield a random selection of patrol officers or perspectives. Existing research also suggests that the findings of this study are likely only generalizable to police–civilian interactions that present modest to no risk to officers (Owens et al., 2018). In spite of these limitations, this study illuminates important directions for future research which should further examine the reciprocal relationship between officer and community well-being.

Recurrent discussions of police reform position police officers and the communities they police on opposite and antagonistic sides. This study shows that a democratic approach to policing, that is, policing that emphasizes fair, transparent, and respectful treatment of civilians and non-enforcement opportunities for community engagement, is as positive for officers as it is for the communities they police and the achievement of public safety. Embedded in the officers’ narratives presented here is a call for a different kind of police work or what scholars from the Center for Policing and Yale Justice Collaboratory describe as a reimagining of public safety (Goff et al., 2019). One interpretation of these findings would be as evidence for increased trainings on procedural justice or for more cops in communities. However, if policing continues to be defined solely in terms of crime reduction and deterrence, this institution will continue to harm the officers that engage in the work and the communities they police. Instead, these narratives reveal that the healthiest parts of their job are in fact not aspects traditionally equated with law enforcement; they are the parts that center empathy, care, and mutuality. That officers’ support for these elements predicts better health outcomes should come as no surprise. Police are given the impossible task of “controlling” social problems created by an ecosystem of failing institutions, and too often, they are provided one tool to do so, as one officer described, “the hammer of the law.” Almost all of the officers interviewed in this study saw themselves as protectors and civil servants. The current construction of policing disallows for the realization of those goals, negatively impacting officers’ mental and emotional wellness. In this department, we see the beginnings of a new model, one that moves toward an understanding of public safety rooted in dignity, cooperation, and community participation.

## Data Availability Statement

In order to maintain the confidentiality and security of police data, CPE plans on sharing data in the following manner. All data will be stored on a secure server, and because we cannot make the database broadly accessible while simultaneously maintaining police department confidentiality, outside researchers will apply to analyze our data. Researchers can propose research ideas to CPE via an application process. Once applications have moved beyond the initial screening, CPE staff will provide outside researchers with a dummy variable set upon which the outside researchers will conduct their analyses. The outside researchers will then submit their syntax back to the project staff who will run that syntax on the actual data set. Finally, project staff will screen the final analyses before returning the outcomes to the outside researchers in order to ensure that no one can identify an individual department. This is a labor-intensive—but necessary—process by which the database can be a tool for all researchers interested in the influence of policing on society. Requests to access the datasets should be directed to: The Center for Policing Equity at coordinator@policingequity.org.

## Ethics Statement

The studies involving human participants were reviewed and approved by Office for Protection of Human Subjects (OPHS) UC Berkeley Protocol: 2018-04-11001. The patients/participants provided their written informed consent to participate in this study.

## Author Contributions

The author confirms being the sole contributor of this work and has approved it for publication.

## Conflict of Interest

The author declares that the research was conducted in the absence of any commercial or financial relationships that could be construed as a potential conflict of interest.

## Appendix A: Semi-Structured Interview Protocol

### Background

1. How did you come to police work? What motivated you to become a police officer?
2. What does being a police officer mean to you?
3. What was the process of becoming a police officer like? Has it changed anything about you?
4. What is the most important part about police work?
5. Tell me about a typical shift?
6. Tell me about your last shift?

### Non-compliance

1. How do you define non-compliance?
2. How often are the people you interact with non- compliant?
3. Tell me about a difficult incident where someone was being non-compliant?
  (a) What made it difficult?
  (b) What led up to this incident?
  (c) How did it resolve? Were there other people around? Did you have or call for back-up? Was it a discretionary stop or call for service? What kind of call? Time of day? Location?

### De-Escalation

1. How do you define de-escalation?
2. I’ve heard officers talk about situations going “sideways.” What does that mean to you? How do you know if an interaction is going sideways? Did you use de-escalation techniques? Which ones?
  (a) Were there other people around? Did you have or call for back-up? Was it a discretionary stop or call for service? What kind of call? Time of day? Location?
3. What have your de-escalation trainings been like? Which elements have been the most useful for you on the street?

### Safety

1. Tell me about the time you felt you the least safe and why?

### Us Versus Them

1. What does the phrase, “us vs them,” mean to you?
2. Have you ever experienced those kinds of feelings in your own work? Can you describe those feelings or an incident that you think might exemplify your understanding of the phrase?
3. Do you think the “us verus them” characterization (whether they report having experienced it or not), is an issue that affects officers’ ability to do their jobs? How/why? How/why not?

### Demographics

1. How do you identify? Age, upbringing, nationality, rank, years on, shift, assignment/beat, political affiliation.

### Conclusion

1. Is there anything else that we didn’t cover that you would like to share with me?
